# London Editors and London Hospitals

**Published:** 1893-08-05

**Authors:** 


### The Hospital

An Institutional Journal of

Science, Medicine, Nursing, and Philanthropy.

*For Hospitals, Asylums, Infirmaries, Dispensaries, Medical Practitioners, Students, Nurses, and the Charitable Public.*

Vol. XIV.—No. 358.] Aug. 5, 1893.

### London Editors and London Hospitals.

The press, it is sometimes said, has taken the place of the pulpit in the modern world. If that be so, it is desirable that the modern world should give a good deal of its best attention to newspaper editors. We Britons are a tough generation. Our forefathers took the measure of feudalism, and finding it overbearing and wanting in "sweet reasonableness," they stamped their heels upon it, and extinguished it for ever. Similarly the Stuart kings, notwithstanding that one of them wrote a "Counterblast to Tobacco," and another actually had the temerity and the sublime presence of mind to throw the Great Seal into the Thames, were made to feel the weight of the Englishman's hand, and the contempt of his robust mind, and to yield their imbecility to his sense and resolution We have not stamped out feudalism and reduced the "divine right" to human reasonableness in order to be finally manipulated into mental slavery by the pen and the ink-pot.

We have all seen how the editor of the *Pall Mall Gazette* has actually behaved in the affair of the London Hospital. Let us now consider how he might have behaved, and how he would have behaved if he had been endowed with the large common-sense of a Shakespeare, or the cultured reasonableness of a Matthew Arnold. We purposely compare him with some of our typical English writers, because that is the only standard of comparison with which the best class of English editors would desire to be compared. If we were to compare them with American journalists of the sensation-loving class they would have the best of reasons for thinking we did not desire to value them at their true worth. How, then, might the editor of the *Pall Mall Gazette* have behaved towards the London Hospital if he had desired to serve the cause of the suffering poor and the objects of the higher medical progress? Let us suppose that he has sent his "special commissioner" down to the London Hospital! She returns, and makes 12 charges. Of those 8 are entirely false, as our own special commissioner demonstrates at pp. 301 *et seq.* of our present issue; 2 are true; and 2 are misleading. But even supposing all the charges had been true, they were not serious charges. If a little bread had been wasted, or a few probationers had been occasionally a little overworked, there was nothing deadly in that. The editor of the *Pall Mall* is not a woman to shriek at the sight of a mouse. Would he not, if he had been a robust-minded, wholesome-hearted, Shakespearian Englishman, have written a private letter or visited the London Hospital to find out for himself whether or not the facts were as stated; and if he had found them so, would he not have endeavoured to get them remedied by private remonstrance first of all, rather than by shrieking out at the top of his newspaper's voice that the London Hospital was a villainous institution.

We have already proved how grievous is the libel which the *Pall Mall Gazette* has published on our greatest and best managed voluntary hospital. In the *Pall Mall* of the 2nd inst. that libel is aggravated by the publication of a leading article which treats the emphatic denial of the truth of these articles by Sir Andrew Clark, Mr. T. H. Buxton, the Treasurer, and Mr. John H. Hale, the Chairman of the London Hospital, as no answer to its charges, and denominates their statement as "lamentably weak and utterly inconclusive." The *Pall Mall Gazette* further describes the proof they give of a palpable falsehood with which its commissioner commenced the attack as a "red herring across the scent." There is no apology or withdrawal of these false statements, not even an expression of regret that they should have appeared in the columns of the *Pall Mall Gazette*. Now, Sir Andrew Clark and his colleagues are men of the highest honour, who have devoted very many years to the management and care of the patients in the London Hospital, and who know from personal experience and investigation that the articles in the *Pall Mall Gazette* are contrary to truth and fact. Yet the editor ventures to demand that these gentlemen and the London Hospital who, as our commissioner shows elsewhere, are in the right, should enter upon their defence before an impartial committee. No doubt this is a desperately bold step, but it is idle to spread a net like this in the sight of any bird as the editor must know full well.

It is, of course, the *Pall Mall Gazette*, not the London Hospital, which is on its trial at the present moment. There has already been an inquiry into these charges, or most of them, before an impartial committee, that of the House of Lords, which summoned witnesses, and examined and cross-examined them on oath. As the result of this inquiry, the Lords Committee found, though the editor of the *Pall Mall Gazette* has carefully avoided stating this, that the London Hospital was, in their opinion, "deserving of the greatest measure of charitable support." In these circumstances the only inquiry that can now take place must be held before a judge of the High Court, where the defendant will be. not the London Hospital, but the editor of the *Pall Mall Gazette.* He has been misled, no doubt, by a small clique, not, as he states, by "a strong minority of the Governors," which latter has no existence except in his imagination, as he will find to his cost. Despite public and private information of undoubted accuracy, despite the proofs in his possession of the unreliability of his special correspondent on a vital point, he still persists that the best administered hospital in this country, and probably in the world, is "suspect," and that "the specific charges," all evidence to the contrary notwithstanding, are true in substance and in fact. The editor further announces his intention of giving his commissioner and other anonymous correspondents the use of the columns of the *Pall Mall Gazette* for further statements of a similar character to those already published. Very well. We rejoice that he should thus emphasize and increase the responsibility for which he will no doubt pay adequate damages in the end, because the reality of the maliciousness of the small clique which now holds his conscience will then be proved up to the hilt. Nothing short of an action at law will put an end to this scandulous abuse of the position of an editor in charge of a great journal, and the sooner steps are taken to bring the case into Court the better it will be for the undoubtedly great issues which will thus be determined in the interests of truth and justice.

But the present controversy has wider bearings. It reveals that the questions which the responsible newspaper editor has to put to himself, are—Is "copy" which will sell the day's issue the paramount consideration, or is the well-being of the country of its institutions, and of all the classes in it? There are in England two classes of newspapers—the one class is right minded, loyal to the noble public traditions of a great country, truthful, and dignified; the other is the sensational copy-at-any-price class—the class which sets its own commercial interests before the highest claims of public well-being, public honour, and public duty. To which of these two classes does the *Pall Mall Gazette* now belong? Under Mr. John Morley it belonged to the former class; even under excitable and excitement-loving Mr. Stead it still conspicuously belonged to the former class. But under its present editor, the *Pall Mall Gazette*, if it is to be judged by its articles on the London Hospital, has deserted the ranks of honour-loving journalism.

A London editor has done his best to ruin the London Hospital. It is our hope that by means of other London editors that hospital may be saved from ruin. If the hospital had been a man or a woman who had been thus maliciously assailed, a hundred editors would have rushed to an investigation; and a newspaper war would have brought the truth to light. Are there no morning or evening newspapers which have chivalrous special commissioners who may be sent down to the London Hospital to investigate the truth on the spot? That is what we ourselves have done. In defending the London Hospital "we speak what we know, and testify that we have seen." We make an appeal to every editor in London on behalf of London's largest hospital, and still more urgently on behalf of the tens of thousands at the East-end to whom that hospital is a refuge from life's fiercest storms and a daily salvation from death. Where are the "special commissioners" who will judge between the youthful editor of the *Pall Mall Gazette* and the greatest, most useful, and most entirely indispensable of all our medical charities?

